# Early postnatal nutrition after preterm birth and cardiometabolic risk factors in young adulthood

**DOI:** 10.1371/journal.pone.0209404

**Published:** 2018-12-28

**Authors:** Julia Suikkanen, Hanna-Maria Matinolli, Johan G. Eriksson, Anna-Liisa Järvenpää, Sture Andersson, Eero Kajantie, Petteri Hovi

**Affiliations:** 1 Department of Public Health Solutions, National Institute for Health and Welfare, Helsinki, Finland; 2 Children’s Hospital, Pediatric Research Center, University of Helsinki and Helsinki University Hospital, Helsinki, Finland; 3 Department of Nursing Science, University of Turku, Turku, Finland; 4 Department of General Practice and Primary Health Care, University of Helsinki and Helsinki University Hospital, Helsinki, Finland; 5 Folkhälsan Research Center, Helsinki, Finland; 6 PEDEGO Research Unit, MRC Oulu, Oulu University Hospital and University of Oulu, Oulu, Finland; 7 Department of Clinical and Molecular Medicine, Norwegian University of Science and Technology, Trondheim, Norway; Universidad Miguel Hernandez de Elche, SPAIN

## Abstract

**Objectives:**

Adults born preterm at very low birthweight (VLBW; <1500 g) have a non-optimal cardiometabolic risk factor profile. Since higher protein intake during the first weeks of life predicted a healthier body composition in adulthood in our previous studies, we hypothesized that it would also predict a favorable cardiometabolic profile.

**Study design:**

The Helsinki Study of VLBW Adults includes 166 VLBW and preterm infants born between 1978 and 1985. We collected postnatal nutrition data among 125 unimpaired subjects, who attended two study visits at the mean ages of 22.5 and 25.1 years. We evaluated the effects of energy and macronutrient intakes during the first three 3-week periods of life on key cardiometabolic risk factors with multiple linear regression models. We also report results adjusted for prenatal, postnatal and adult characteristics.

**Results:**

Macronutrient and energy intakes were not associated with blood pressure, heart rate, or lipid levels in adulthood. Intakes were neither associated with fasting glucose or most other markers of glucose metabolism. An exception was that the first-three-weeks-of-life intakes predicted higher fasting insulin levels: 1 g/kg/day higher protein intake by 37.6% (95% CI: 8.0%, 75.2%), and 10 kcal/kg/day higher energy intake by 8.6% (2.6%, 14.9%), when adjusted for sex and age. These early intakes similarly predicted the adult homeostasis model assessment index. Further adjustments strengthened these findings.

**Conclusions:**

Among VLBW infants with relatively low early energy intake, early macronutrient and energy intakes were unrelated to blood pressure, lipid levels and intravenous glucose tolerance test results. Contrary to our hypothesis, a higher macronutrient intake during the first three weeks of life predicted higher fasting insulin concentration in young adulthood.

## Introduction

Globally, neonatal care has greatly improved in recent decades. Accordingly, the prognosis of preterm (<37 gestational weeks) and very low birthweight (VLBW; <1500 g) infants has improved considerably. In high-income countries, the first generation of adults born VLBW who have experienced modern neonatal care are now in their thirties. Preterm birth predisposes to many health problems in adulthood, including a worse cardiometabolic risk factor profile. [1–5] The mechanisms behind the risks remain, partly unknown. The adverse environment that preterm infants face during the postnatal period may have long-lasting effects on health in adulthood, although direct evidence is scarce. [6–8]

In full-term infants, higher protein intake (high-protein formula) during early life is associated with higher BMI as well as a higher risk for obesity at school age. [9] The current recommendations of protein intakes for preterm infants exceed those for term infants (Table 1). [10] Studies on preterm infants’ early nutrition and its long-term effects on cardiometabolic health, however, are limited and contradictory, [11] with most only including effects during infancy and childhood. Higher protein intake after term age was beneficial, as assessed during infancy, predicting lower fat mass or body fat percentage. [12,13] Higher carbohydrate intake during the first month, by contrast, was not beneficial: it was associated with higher weight in childhood. [7] Neonatal nutrition was unrelated to findings from an intravenous glucose tolerance test at age 4 to 10 years. [7] A handful of studies presenting early nutrition of preterm infants and later outcomes have extended to adolescence. In a study of 60 preterm individuals born < 35 gestational weeks, daily energy intakes of more than 70 kcal/kg during the first two weeks of life predicted taller adolescent height and higher weight, but had no effect on blood pressure or body fat percentage. [8] A series of randomized trials comparing breastmilk, which contains low amounts of protein, with regular or preterm formula among infants born preterm and <1850 g have shown an association between breastmilk and lower mean arterial blood pressure, lower fasting 32–33 split pro-insulin concentration, and lower LDL/HDL cholesterol ratio at teenage age. [14–16] However, it is unclear whether these associations are due to breastmilk *per se* or some other characteristic of early feeding, such as low protein content.

**Table 1:** Intakes of energy and macronutrients during the first nine weeks of life.

		Energy, kcal/kg/d			Protein,	Fat,		Carbohydrate,
	n	Total intake	From human milk		g/kg/d	g/kg/d		g/kg/d
Weeks 1–3	125	94.2 (15.6)	78.2 (24.0), 83.1%		1.4 (0.4)*	4.3 (1.1)		11.1 (1.3)
Weeks 4–6	121	119 (14.7)	109 (22.1), 91.2%		1.9 (0.4)*	5.9 (0.4)		12.4 (1.3)
Weeks 7–9	112	124 (13.3)	109 (24.7), 87.9%		2.1 (0.5)*	6.1 (0.9)		12.9 (1.3)
Current recommendations		110–135			1) 4.0–4.4 2) 3.5–4.0	4.8–6.6		11.6–13.2

Values are means (standard deviations).

Current published recommendations, minimum and maximum. [10]

1, 2) Recommendations for protein intake when infant weight is 1) <1kg, 2)1.0–1.8kg.

*The ranges of protein intakes were 0.5–2.7g/kg/d (weeks 1–3), 1.0–3.3 g/kg/d (weeks 4–6), and 0.9–3.7 g/kg/d (weeks 7–9).

Very few studies have extended the follow-up of preterm infants to adult life. In the Helsinki Study of Very Low Birthweight Adults (HeSVA), from which the current analysis is performed, a higher early protein, fat, and energy intake during the first weeks of life predicted higher lean body mass and better cognitive function in young adult life. [6,17] By today’s standards, these infants received low although variable amounts of protein, and thus constituted a natural experiment regarding the long-term effects of protein intake (Table 1). We hypothesized that among preterm subjects born at VLBW and characterized by relatively poor nutrition, higher intakes of macronutrients and energy during the first weeks of life would predict a more optimal cardiometabolic profile in young adult life. We expected this difference to be largest among those most premature: those born extremely preterm (< 28 gestational weeks) or small for gestational age (SGA).

## Methods

### Study design

The original HeSVA cohort consisted of 335 VLBW (<1500 g birthweight) and preterm (<37 gestational weeks) infants who received intensive care treatment at Children’s Hospital of Helsinki University Central Hospital between 1978 and 1985. [1] A full-term and appropriate-for-gestational age (AGA) comparison group was selected from birth hospital records when the study cohort reached young adulthood, but were not included in the current analysis. We invited 255 VLBW subjects living in the greater Helsinki area for a clinical examination as young adults, and 166 VLBW subjects participated. We were able to collect the full first nine weeks of nutrition data for 141 VLBW subjects. [6] We excluded 14 subjects with a neurosensory impairment (cerebral palsy, mental retardation, and blindness), 1 with hypothyroidism, and 1 who could not maintain an 8 hour fast before laboratory testing because these conditions can affect body composition and metabolism through distinct pathways; 125 VLBW subjects remained in the analysis. The Ethics Committee of Children and Adolescents’ Diseases and Psychiatry at the Helsinki University Central Hospital, Finland, approved the study plan, and all participants gave their written informed consent.

We extracted information about pregnancy and the neonatal period from hospital and maternity clinic records. [1,6] As previously described, [6] the protocol for feeding VLBW infants in Finland at the time the cohort was born, was to begin with pooled, banked, and pasteurized human milk via nasogastric tube during the first or second day of life. Daily milk intake was increased according to individual tolerance until 200 mL/kg/d (mother’s own milk and, if needed, banked human milk), at which it was sustained until discharge. Calcium, phosphate and multivitamin supplementation were used throughout hospitalization. The content of the mother’s own breastmilk was approximated based on figures by Anderson et al. and the content of banked human milk was measured at the same hospital during the 1980s for another study. [18–20] Of the participants, 22% (n = 28) also received fortifiers of preterm formula during the first 9 weeks, but only 1 during the first 3 weeks. Additionally, 35% (n = 44) of the participants also received intravenous fluids with glucose, 17% (n = 22) received parenteral amino acids, and 15% (n = 19) received parenteral lipids. Even 59 (47%) of the participants received parenteral nutrition during the first 9 weeks of life, only 16 (13%) received it for more than 1 day. From daily registrations of milk, fortifier and supplement intakes, we calculated daily nutrient intakes for macronutrients (protein, fat, and carbohydrates), and energy. [6] We restricted the nutrition analysis to the first 9 weeks of life because of the timing of hospital discharge. Details of specific nutrient-intake calculations have been previously described. [6] Growth information (weight, length, and head circumference) was collected from hospital and child welfare clinic records. When needed, we interpolated daily values from closest measurements. SGA was defined as less than -2 standard deviation score (SD) birth weight according to Finnish newborn growth charts. [21] Postnatal SD scores were also calculated according to Finnish newborn growth charts in relation to gestational age.

### Clinical measurements

At a mean age of 22.5 (SD 2.1) years, the participants completed detailed questionnaire on socioeconomic status, medical history, use of medications, and smoking status. After at least an 8-hour period of fasting, the participants’ blood pressure, heart rate, weight, height, and hip and waist circumference were measured and BMI was calculated. We drew blood for laboratory examinations of lipids (cholesterol, HDL cholesterol, and triglycerides). At oral glucose tolerance test both glucose and insulin levels were measured. We also calculated the homeostasis model assessment to quantifying insulin resistance (HOMA-IR). [22] For the entire study cohort, mean values, comparisons and details are described elsewhere. [1] For all subjects in the current analysis, we also measured body composition with whole-body DXA (Dual energy X-ray Absorptiometry). [23] At a mean age of 25.1 (SD, 2.1) years, a subgroup of 78 of the 125 underwent a standard 14-sample intravenous glucose tolerance test. [24] As the main outcome variables we used insulin sensitivity index (Si, quantifying the capacity of insulin to promote glucose disposal), first-phase insulin secretion (AIR, calculated between baseline and 10 min as the insulin area under the curve (AUC) over baseline), and disposition index (DI, calculated as AIR*Si, an estimate of beta cell function). [24]

### Statistical analysis

All statistical analyses were performed with IBM SPSS Statistics for Windows 23. Differences were considered statistically significant at a p-value < 0.05. We first ran the analyses in our study group of 125, then separately among those extremely preterm (born <28 gestational weeks, n = 35), ≥28 gestational weeks (n = 90), SGA (n = 37), and AGA (n = 88). Our linear regression models investigated the effects of protein, fat, carbohydrate and energy intakes in the neonatal period on the adult cardiometabolic risk factors. These risk factors were: fasting glucose and insulin concentrations, HOMA-IR, glucose and insulin concentrations after a 2-hour oral glucose tolerance test, insulin sensitivity index, first-phase insulin secretion and disposition index, systolic and diastolic blood pressure, heart rate, total cholesterol, HDL cholesterol, and triglycerides. We ran a separate analysis for the mean intake of total energy and each macronutrient during three time periods (the first 3 weeks of life, 4–6 weeks of life, and 7–9 weeks of life). We analyzed insulin and glucose concentrations and HOMA-IR as log transformations to attain normality of the residuals. After analysis, the betas were back-transformed, so the results are expressed in percent changes.

As the effect of early nutrition to later cardiometabolic outcomes is at least partly conducted via early growth (S1 Table), we also measured early growth as gain in weight (grams), length (SD units), and head circumference (SD units). As a complementary analysis, we studied the effect of early growth in the first 3 weeks and 4–6 weeks of life on the cardiometabolic risk factors.

We adjusted the regression models for sex and age at clinical examination (Model 1). Model 2 also included gestational age and birthweight SD score, highest parental education, maternal smoking during pregnancy (from the hospital records), prenatal maternal preeclampsia, postnatal characteristics, adult body fat percentage, leisure-time exercise intensity, and smoking status. Postnatal characteristics, all prospectively confirmed by a neonatologist, were neonatal treatment with ventilator (in days), bronchopulmonary dysplasia, culture-positive septicemia, and persistent ductus arteriosus. When analyzing effects of growth in body weight and length, Model 2 additionally included age at neonatal minimum weight. The covariates were selected because of a priori expectations. We excluded adult lean body mass (measured with whole-body DXA) from the final models since it changed estimates only marginally. Early nutrient intakes and sex did not have a significant interaction, so sex-specific analyses were not performed. As a complementary analysis, we performed the main analysis excluding multiple pregnancies (n = 21, 17%), and excluding those who received parenteral nutrition for more than one day (n = 16, 13%).

## Results

### Characteristics of the subjects

Our study population consisted of 125 subjects; the average gestational age was 29 (SD 2.2) weeks with range of 24.1 to 35.6 weeks, and the average birth weight 1100 g (SD 220 g) (Table 2).

**Table 2:** Characteristics of the young adults born at VLBW.

Neonatal characteristics	Mean (SD) or n (%)	Missing values, n
Women	72 (58%)	
Gestational age, wk	29.0 (2.2)	
Birth weight, g	1101 (217)	
Birth weight, SD score	-1.2 (1.5)	
Length at birth, cm	36.8 (2.4)	1
Small for gestational age	37 (30%)	
Multiple pregnancy	21 (17%)	
Maternal smoking during pregnancy	24 (19%)	
Maternal preeclampsia	23 (18%)	
Maternal chorioamnionitis	8 (6%)	
Premature rupture of membranes	13 (10%)	
Received supplemental oxygen	116 (93%)	5
Supplemental oxygen, d	15 (4; 35)*	5
Received mechanical ventilation	90 (72%)	2
Mechanical ventilation, d	6 (0; 17)*	2
Bronchopulmonary dysplasia (criteria by [25])**	24 (19%)	
Sepsis	8 (6%)	3
Persistent ductus arteriosus	36 (29%)	2
Weight, SD score at term age	-2.9 (0.9)	14
**In clinical study**		
Age, years	22.5 (2.1)	
Height (women), cm	163 (7.2)	
Height (men), cm	175 (7.1)	
BMI (women), kg/m^2^	22.3 (3.9)	
BMI (men), kg/m^2^	21.7 (3.0)	
Waist circumference (women), cm	78.0 (10.0)	
Waist circumference (men), cm	81.3 (8.2)	
Hip circumference (women), cm	96.6 (8.4)	
Hip circumference (men), cm	93.3 (6.2)	
Percentage body fat (women), %	31.5 (6.0)	5
Percentage body fat (men), %	19.0 (5.0)	4
Lean mass (women), kg	39.9 (5.6)	5
Lean mass (men), kg	53.6 (7.8)	4
Systolic blood pressure, mmHg	122 (14.3)	
Diastolic blood pressure, mmHg	79.1 (8.9)	
Heart rate (beats per minute)	75.7 (12.8)	
Fasting glucose, mmol/L	4.7 (0.4)	
Glucose after 2 hour glucose tolerance test, mmol/L	5.4 (1.2)	
Fasting insulin, mU/L	6.3 (3.8)	
Insulin level after 2 hour glucose tolerance test, mU/L	40.7 (34.0)	
Total cholesterol, mmol/l	4.5 (0.8)	
HDL cholesterol, mmol/l	1.7 (0.4)	
Triglyserides, mmol/l	1.1 (0.5)	
Smoking	32 (26%)	3
Leisure-time physical activity		
Competing	5 (4%)	3
Brisk >3h/wk	30 (24%)	
Light >4h/wk	42 (34%)	
Not much	45 (36%)	
Parental education		
Elementary	12 (10%)	3
Middle school	24 (19%)	
High school	51 (41%)	
University	35 (28%)	

n = 125 unless values were missing

*median (25th; 75th percentiles)

** The diagnosis of bronchopulmonary dysplasia was set by a neonatologist according to Northway criteria [25].

### Early nutrition

Mean nutrient intakes were lower than current recommendations (Table 1). All but 6 subjects achieved the recommended energy intake during the first 9 weeks and the recommended carbohydrate and fat intakes after 4 weeks, but only 1 out of 125 subjects reached sufficient protein intake during the first 9 weeks (Table 1). During weeks 1 to 9 of life, mean energy intake from human milk was 83.1–91.2% (Table 1). Since the subjects’ early nutrition was meagre, they also grew sub-optimally in body size (Table 3).

**Table 3:** Weight, length and head circumference at birth and at 3 and 6 weeks of age.

	n	g or cm	SDS*
Birth weight, g	125	1120 (220)	-1.2 (1.5)
Weight at 3 weeks of age, g	121	1160 (264)	-3.3 (0.9)
Weight at 6 weeks of age, g	115	1510 (342)	-3.4 (0.8)
Birth length, cm	125	36.9 (2.4)	-1.3 (1.7)
Length at 3 weeks of age, cm	120	38.9 (2.5)	-2.5 (1.5)
Length at 6 weeks of age, cm	113	41.0 (2.6)	-3.1 (1.3)
Birth head circumference, cm	125	26.2 (1.9)	-1.1 (1.4)
Head at 3 weeks of age, cm	119	26.9 (2.1)	-2.7 (1.0)
Head at 6 weeks of age, cm	113	29.3 (2.3)	-2.5 (1.5)

Values are given as means (SDs).

*Standard deviation scores (SDS) are calculated from Finnish newborn growth charts in relation to gestational age [21].

### Early macronutrient intakes in relation to glucose tolerance and insulin sensitivity in adulthood

Higher average daily intakes of energy, carbohydrate, fat, and especially protein during the first 3 weeks of life predicted higher fasting insulin concentrations in young adulthood (Table 4). A 1 g/kg/day higher protein intake predicted a 37.6% higher fasting insulin level (95% Confidence interval (CI): 8.0%, 75.2%, p = 0.01, Model 1) and a 43.3% higher HOMA-IR (95% CI: 11.0%, 84.9%, p = 0.006, Model 1). The results were little changed when adjusted for perinatal and postnatal characteristics. In analyses of subsets of the cohort, the positive association between early nutrient and energy intake and fasting insulin concentration and HOMA-IR was strongest in the 35 participants born extremely preterm, but was absent among those 90 born at or after 28 weeks (p values for interactions <0.05). Among those born extremely preterm, a 1 g/kg/day higher protein intake predicted a 178% higher fasting insulin level (95% CI: 65.7%, 365%, p < 0.001, Model 1) and a 194% higher HOMA-IR (95% CI: 80.1%, 379%, p < 0.001, Model 1). Effect differences between AGA and SGA subgroups did not reach statistical significance (p for interaction 0.12 with fasting insulin). Nutrient intakes during weeks 4 to 6 and 7 to 9 were not associated with fasting insulin or HOMA-IR concentrations.

**Table 4:** Effect of the first 3 weeks of life energy and macronutrient intake of VLBW infants on a 2-hour glucose tolerance test in young adult life, by separate linear regression models.

		Fasting insulin, %	p	HOMA-IR, %*	p	2-hour insulin, %	p	Fasting Glucose, %	p	2-hour glucose, %	p
**Energy, 10kcal/kg/d**											
Model 1	8.6 (2.6, 14.9)		0.005	9.4 (3.0, 16.1)	0.004	2.7 (-4.5, 10.3)	0.47	0.8 (-0.1, 1.7)	0.08	-0.5 (-3.0, 2.2)	0.73
Model 2 (Full model)	9.7 (2.9, 17.0)		0.005	10.4 (3.2, 18.2)	0.005	5.4 (-3.2, 14.7)	0.22	0.7 (-0.3, 1.7)	0.19	0.6 (-2.4, 3.7)	0.69
<28 weeks (Model 1)	21.8 (7.6, 38.0)		<0.001	23.8 (10.2, 39.0)	0.001	11.4 (-3.1, 28.2)	0.12	2.1 (0.4, 3.8)	0.04	0.8 (-5.0, 7.0)	0.78
≥28 weeks (Model 1)	2.7 (-3.9, 9.7)		0.42	3.0 (-4.0, 10,4)	0.41	-1.4 (-9.8, 7.8)	0.76	0.3 (-0.8, 1.4)	0.58	-1.4 (-4.4, 1.6)	0.35
**Protein, g/kg/d**											
Model 1	37.6 (8.0, 75.2)		0.01	43.3 (11.0, 84.9)	0.006	7.1 (-21.1, 45.2)	0.66	4.3 (0.6, 8.3)	0.02	0.0 (-3.7, 3.8)	0.999
Model 2 (Full model)	50.6 (16.1, 95.4)		0.002	54.9 (17.7, 103.8)	0.002	30.8 (-7.2, 84.4)	0.12	3.1 (-1.1, 7.5)	0.15	7.6 (-4.8, 21.6)	0.24
<28 weeks (Model 1)	177.6 (65.7, 365.0)		<0.001	193.8 (80.1, 379.3)	<0.001	115.7 (19.7, 288.8)	0.01	11.7 (4.1, 19.9)	0.003	18.8 (-8.5, 54.2)	0.19
≥28 weeks (Model 1)	4.3 (-20.7, 37.2)		0.76	6.2 (-20.5, 41.9)	0.68	-17.7 (-42.9, 18.6)	0.29	2.2 (-2.3, 6.8)	0.34	-6.8 (-17.8, 5.8)	0.27
**Fat, g/kg/d**											
Model 1	8.5 (-0.1, 17.9)		0.08	9.4 (0.2, 19.4)	0.05	2.1 (-7.9, 13.2)	0.69	0.8 (-0.5, 2.1)	0.20	-0.9 (-4.0, 2.2)	0.99
Model 2 (Full model)	9.6 (0.1, 20.1)		0.05	10.1 (0.0, 21.2)	0.05	5.8 (-6.0, 19.0)	0.35	0.4 (-1.0, 1.9)	0.55	1.5 (-2.7, 5.9)	0.48
<28 weeks (Model 1)	29.8 (3.9, 62.1)		0.02	193.8 (80.1, 379.3)	0.008	25.7 (-0.1, 58.2)	0.05	2.1 (-0.8, 5.2)	0.16	3.7 (-6.1, 14.6)	0.46
≥28 weeks (Model 1)	2.0 (-6.8, 11.7)		0.67	2.5 (-6.9, 12.7)	0.61	-4.3 (-15.2, 8.1)	0.48	0.50 (-1.0, 2.0)	0.50	-1.5 (-5.5, 2.7)	0.47
**Carbohydrate, g/kg/d**											
Model 1	8.8 (1.5, 16.5)		0.02	9.4 (1.7, 17.6)	0.02	0.3 (-8.0, 9.3)	0.95	0.6 (-0.4, 1.7)	0.23	-0.9 (-4.0, 2.2)	0.56
Model 2 (Full model)	10.1 (2.6, 18.0)		0.008	11.1 (3.2, 19.6)	0.006	2.4 (-6.6, 12.3)	0.61	1.0 (-0.1, 2.1)	0.08	-0.1 (-3.3, 3.3)	0.97
<28 weeks (Model 1)	16.6 (1.5, 34.0)		0.03	20.1 (4.3, 38.2)	0.01	2.6 (-11.8, 19.4)	0.73	1.8 (0.0, 3.6)	0.14	-0.9 (-6.9, 5.5)	0.77
≥28 weeks (Model 1)	2.5 (-5.8, 11.6)		0.56	2.5 (-6.3, 12.1)	0.59	-2.2 (-12.7, 9.6)	0.70	0.1 (-1.3, 1.5)	0.92	-1.4 (-5.2, 2.6)	0.48

The values are unstandardized regression coefficients, B (95% CI). Model 1 adjusted for sex and age. Model 2 additionally adjusted for gestational age, birth weight SD score, highest parental education, maternal smoking during pregnancy, maternal preeclampsia, postnatal characteristics (neonatal exposure of treatment with ventilator (days), bronchopulmonary dysplasia, septicemia and persistent ductus arteriosus), adult body fat percentage, leisure-time exercise intensity and smoking status.

* HOMA-IR is homeostasis model assessment to quantifying insulin resistance

In addition to association of early protein intake with higher fasting insulin concentrations, higher protein intake during the first 3 weeks of life was associated with higher fasting glucose concentration. However, this attenuated to non-significance after adjustment with perinatal and postnatal characteristics. This association was also stronger in the extremely preterm subgroup, but was not observed in the ≥28-gestational-week subgroup (p for interaction 0.035).

Early nutrition showed no associations with glucose or insulin concentrations at the end of the 2-hour oral glucose tolerance test. We also performed an intravenous glucose tolerance test on a subgroup of 78 individuals (mean gestational age at birth 29.1 weeks). Early nutrition showed no association with insulin sensitivity index, first-phase insulin secretion or their product, disposition index (Table 5).

**Table 5:** Effect of the first 3 weeks of life energy and macronutrient intake of VLBW infants on their A) blood pressures and heart rate, B) insulin sensitivity values and C) lipid concentrations in young adult life, by separate linear regression models.

**A**						
**Energy, 10 kcal/kg/d**	**Systolic blood pressure, mmHg**	**p**	**Diastolic blood pressure, mmHg**	**p**	**Heart rate, beats per minute**	**p**
Model 1	0.15 (-1.37, 1.67)	0.85	-0.40 (-1.41, 0.62)	0.44	-1.09 (-2.57, 0.38)	0.14
Model 2	0.09 (-1.64, 1.81)	0.92	-0.24 (-1.37, 0.89)	0.68	-1.36 (-3.14, 0.42)	0.13
**Protein, g/kg/d**						
Model 1	3.83 (-2.57, 10.23)	0.24	0.95 (-3.35, 5.24)	0.66	-2.37 (-8.66, 3.91)	0.46
Model 2	6.57 (-0.32, 13.46)	0.06	3.54 (-1.01, 8.09)	0.13	-3.64 (-10.93, 3.65)	0.32
**Fat, g/kg/d**						
Model 1	0.65 (-1.53, 2.84)	0.55	-0.10 (-1.56, 1.36)	0.89	-1.71 (-3.82, 0.41)	0.11
Model 2	0.51 (-1.88, 2.91)	0.67	-0.08 (-1.65, 1.50)	0.92	-2.00 (-4.47, 0.47)	0.11
**Carbohydrate, g/kg/d**						
Model 1	-0.66 (-2.47, 1.16)	0.48	-0.73 (-1.93, 0.48)	0.23	-0.91 (-2.68, 0.86)	0.31
Model 2	-0.76 (-2.62, 1.09)	0.42	-0.60 (-1.82, 0.61)	0.33	-0.61 (-2.55, 1.33)	0.53
**B**						
**Energy, 10 kcal/kg/d**	Insulin sensitivity index*	**p**	First-phase insulin secretion*	**p**	Disposition index*	**p**
Model 1	-0.01 (-0.07, 0.06)	0.78	27.05 (-8.91, 63.01)	0.14	0.06 (-0.01, 0.13)	0.08
Model 2	-0.03 (-0.11, 0.04)	0.38	24.91 (-22.27, 72.09)	0.29	0.02 (-0.07, 0.10)	0.71
**Protein, g/kg/d**						
Model 1	-0.00 (-0.29, 0.28)	0.97	80.47 (-78.83, 239.8)	0.31	0.20 (-0.11, 0.51)	0.21
Model 2	-0.20 (-0.52, 0.12)	0.22	68.70 (-128.4, 265.9)	0.49	-0.10 (-0.44, 0.25)	0.58
**Fat, g/kg/d**						
Model 1	-0.02 (-0.11, 0.08)	0.73	36.79 (-14.36, 87.95)	0.16	0.07 (-0.03, 0.17)	0.15
Model 2	-0.05 (-0.16, 0.06)	0.35	22.84 (-42.15, 87.83)	0.48	-0.02 (-0.13, 0.10)	0.78
**Carbohydrate, g/kg/d**						
Model 1	0.02 (-0.06, 0.09)	0.70	16.84 (-27.43, 61.11)	0.45	0.07 (-0.01, 0.16)	0.10
Model 2	0.01 (-0.07, 0.09)	0.82	21.54 (-28.35, 71.44)	0.39	0.06 (-0.02, 0.15)	0.15
**C**						
**Energy, 10 kcal/kg/d**	**Total cholesterol, mmol/L**	**p**	**HDL cholesterol, mmol/L**	**p**	**Triglycerides, mmol/L**	**p**
Model 1	-0.02 (-0.12, 0.07)	0.64	-0.02 (-0.07, 0.03)	0.40	0.05 (-0.01, 0.11)	0.09
Model 2	-0.00 (-0.12, 0.11)	0.99	-0.02 (-0.04, 0.07)	0.51	0.04 (-0.03, 0.11)	0.26
**Protein, g/kg/d**						
Model 1	-0.03 (-0.44, 0.37)	0.87	-0.10 (-0.29, 0.10)	0.34	0.21 (-0.03, 0.45)	0.09
Model 2	0.11 (-0.35, 0.58)	0.63	0.04 (-0.18, 0.26)	0.72	0.22 (-0.05, 0.50)	0.10
**Fat, g/kg/d**						
Model 1	-0.02 (-0.16, 0.12)	0.75	-0.03 (-0.10, 0.04)	0.39	0.08 (0.00, 0.16)	0.05
Model 2	-0.01 (-0.17, 0.15)	0.89	0.01 (-0.07, 0.09)	0.78	0.06 (-0.03, 0.15)	0.20
**Carbohydrate, g/kg/d**						
Model 1	-0.02 (-0.14, 0.09)	0.68	-0.01 (-0.07, 0.04)	0.67	0.01 (-0.06, 0.08)	0.81
Model 2	-0.01 (-0.13, 0.11)	0.86	-0.02 (-0.04, 0.08)	0.54	-0.03 (-0.07, 0.07)	0.98

The values are unstandardized regression coefficients, B (95% CI). Model 1 adjusted for sex and age. Model 2 additionally adjusted for gestational age, birth weight SD score, highest parental education, maternal smoking during pregnancy, maternal preeclampsia, postnatal characteristics (neonatal exposure of treatment with ventilator (days), bronchopulmonary dysplasia, septicemia and persistent ductus arteriosus), adult body fat percentage, leisure-time exercise intensity and smoking status.

*Among the total 125 subjects, an intravenous glucose tolerance test was performed in a subgroup of 78 at a mean age of 25.1 years. Other adulthood measurements used as confounding variables were measured at the first clinical visit (mean age 22.5 years).

In a complimentary analysis excluding multiple pregnancies (n = 21, 17%) or those who received parenteral nutrition for more than one day (n = 16, 13%), fairly similar results appeared than for the whole study group (S2 Table).

### Early macronutrient intakes in relation to young adult blood pressure, heart rate and lipids

Higher energy, protein, fat or carbohydrate intake during the first 3 weeks, 4 to 6 weeks or 7 to 9 weeks of life had no major impact on adult systolic blood pressure, diastolic blood pressure, or heart rate (Table 5). We also could not find any differences in the results between the specific predefined subgroups: those born before or after 28 full gestational weeks, or those born SGA or AGA.

Higher energy, protein, fat, or carbohydrate intakes had no significant impact on adult total cholesterol levels, HDL cholesterol levels, or triglycerides (Table 5).

### Early growth in relation to young adult cardiometabolic risk factors

Weight gain (in grams) and linear growth (in SD units) during the first 3 weeks and weeks 4 to 6 of life were unrelated to all cardiometabolic risk factors assessed (glucose or insulin levels, systolic or diastolic blood pressure, heart rate, and lipids) (S3 Table). Head circumference, however, showed some associations. Weeks 4 to 6 growth in head circumference (in SDs) showed a negative association with systolic blood pressure: 1 SD increase in head growth predicted a 4.78 mmHg lower systolic blood pressure (95% CI: -7.84, -1.72, p = 0.003, Model 1).

## Discussion

Our main finding was that, with the exception of glucose metabolism, the cardiometabolic risk factor profile among adults born preterm at VLBW was not predicted by differences in nutritional intake during the first weeks of life. We have previously shown that low postnatal macronutrient intake predicts lower lean body mass and poorer neurocognitive function. [6,26] However, it had no effect on blood pressure, heart rate, or lipid levels. The only exception was that higher macronutrient intakes during the first 3 weeks of life predicted higher adult fasting insulin levels, especially in those born before 28 gestational weeks. A similar association was seen for fasting plasma glucose when adjusted for sex and age, although it reached statistical significance for protein intake only, and in the final model no associations persisted. No associations were seen with glucose or insulin after an oral glucose load or with specific glucose tolerance markers measured by intravenous glucose tolerance test in the subset that underwent this test. The confidence intervals indicate that we can with reasonable certainty exclude moderate or large effects between early nutrition in VLBW infants and most of the measured cardiometabolic risk factors. For example, the confidence interval for the association between energy intake during the first 3 weeks of life and systolic blood pressure was -1.37 to 1.67 mmHg per 10 kcal/kg/d (corresponding approximately to -0.15 SD to 0.18 SD higher systolic blood pressure per 1 SD higher energy intake).

Our cohort was born between 1978 and 1985. At that time, nutrition guidelines and practices differed considerably from today’s standards. The infants were malnourished especially in terms of protein and grew sub-optimally (Table 3). They were mostly fed with human milk (and calcium, phosphate, and multivitamin supplements) and only a few received preterm fortifiers. The macronutrient content of human milk varies and was not measured directly, but calculated based on figures from previous studies at the same hospital which may have influenced the results. [18–20] Such an estimation assuming equal concents will by definition decrease the within and inter individual variation, as compared to true intakes, and therefore it potentially blunted some existing associations.As to other neonatal treatments, only a handful received antenatal glucocorticoids or surfactant. [27] Partly because of this, our findings cannot be directly applied to preterm infants born today. Our longitudinal study, with substantial variation within low levels of early nutrient intake, rather serves as a unique natural experiment to assess the life course consequences of early postnatal nutrition. Since the cohort subgroups of SGA (n = 37) and extremely preterm (n = 35) were considerably small, these results consequently require further confirmation. The clinical examination was performed at a mean age of 22.5 years. At young adulthood, the possible development toward high cardiovascular risk profiles becomes more easily observable than in children. However, our study outcomes remain limited to risk factors rather than manifest disease endpoints which would occur later in life. One of our strengths was our ability to adjust for a wide range of prenatal and neonatal medical conditions, and we believe it is unlikely that any true moderate association between early nutrient intake and adult cardiometabolic risk factors could be explained or disguised by confounding by such conditions.

Extensive research on the long-term effects of early nutrition in preterm infants comes from a series trials from UK where preterm infants were randomized to receive preterm formula, term formula, or banked breastmilk, as the sole diet or as supplements to mother’s milk until they reached 2000 g of weight or were discharged from the hospital. [14–16,28]. In general, the findings showed few associations between early nutrition and indicators of glucose metabolism. The only exceptions were that in the UK trials at ages 13 to 16 years, those who had been randomized to receive nutrient-enriched preterm formula had higher fasting 32–33 split proinsulin levels, and in our study higher early nutrient intake predicted higher fasting insulin levels in young adulthood. [15]

The same UK trials assessed a number of other cardiometabolic risk factors. Those who had received breastmilk had lower mean arterial blood pressure and lower LDL/HDL ratios; there was no difference between the preterm and term formula groups. [14,16] In our study, variation in early macronutrient intakes was unrelated to blood pressure and plasma lipids. Testing the effects of breastmilk in our study was not feasible since more than 83% of energy during the first 3 weeks (Table 1) came from breastmilk.

Only a few studies have used daily recordings of early nutrient intake. In a New Zealand cohort, 37 children born before 32 gestational weeks underwent an intravenous glucose tolerance test at 4 to 10 years of age. Parallel to our study, insulin sensitivity and secretion were unrelated to early nutrition. As expected, greater weight gain from term age to childhood (4 to 10 years of age) among preterm infants predisposed them to lower insulin sensitivity in childhood. [7] A Canadian study of 61 participants born preterm and <1850 g, examined at a mean age of 14 years, compared those who received >70 kcal/kg/d of energy during the first 2 weeks of life with those whose energy intake was <70 kcal/kg/day. Those who had received more energy were taller and heavier, but had similar blood pressure and percent body fat. [8] In our cohort, higher energy intake did not predict taller or heavier stature, but in parallel with the Canadian study, we found no association with blood pressure and percent body fat. [6] Unfortunately, both the Canadian and New Zealand studies had relatively low power to exclude a clinically meaningful association.

Our cohort is to our knowledge the only cohort which has daily recordings of early nutrient intake of adults born preterm. We have previously shown that higher early protein and energy intakes predict higher adult lean body mass and higher scores in neurocognitive tests. [6,17] Because of these findings, we expected higher early macronutrient intake to predict lower levels of cardiometabolic risk factors, which, however, was not confirmed.

Globally, 11% of infants are born preterm, [29] and survival to adulthood is increasing. Early nutrition is important since it can be modified, especially among very preterm infants who spend their first weeks in the hospital and undergo intensive intravenous or oral nutrition regimens. Our findings suggest that the associations that are seen between higher energy and protein intakes during the first weeks of life and higher lean body mass and cognitive abilities in young adulthood are not reflected in the majority of cardiometabolic risk factors. [6,17] Impaired glucose regulation, however, was related to higher intakes which, if replicated, would support the idea of a tradeoff between cognition and cardiovascular health.

## Conclusion

At considerably low nutrient intake levels, higher nutrient and energy intake among preterm and VLBW infants predicts higher fasting insulin levels in adulthood, but does not associate with other markers of insulin and glucose metabolism, blood pressure, heart rate, or lipid levels.

## Supporting information

S1 Table Early nutrition and growth. Associations between nutrient and energy intakes of very low birth weight infants during the first three weeks of life and weight gain during the same weeks.
(DOC)

S2 Table Sensitivity analysis. Analysis of the first 3 weeks of life energy and macronutrient intakes of those born very low birth weight and cardiometabolic outcomes in adulthood, after exclution of those who recieved parenteral nutrition for more than one day or where born from a multiple pregnancy.
(DOC)

S3 Table Early growth and cardiometabolic outcomes in adulthood. Effect of early growth (weeks 1 to 3 and 4 to 6 of life) of very low birth weight and preterm infants on cardiometabolic outcomes in adulthood.
(DOC)

## Abbreviations

AGA: appropriate for gestational age
HESVA: the Helsinki Study of Very Low Birth Weight Adults
HOMA-IR: homeostasis model assessment to quantifying insulin resistance
SGA: small for gestational age
VLBW: very low birth weight (<1500 g)
