# Supplementary material for: Early postnatal nutrition after preterm birth and cardiometabolic risk factors in young adulthood
Source: PLoS One. 2018 Dec 28;13(12):e0209404. doi: 10.1371/journal.pone.0209404 (PMC6310277; doi:10.1371/journal.pone.0209404)
Supplement: S1 Table — Associations between nutrient and energy intakes of very low birth weight infants during the first three weeks of life and weight gain during the same weeks. (DOC) [file pone.0209404.s001.doc]

**S1 Table.** **Early nutrition and growth.** Associations between nutrient and energy intakes of very low birth weight infants during the first three weeks of life and weight gain during the same weeks. Values are regression coefficients adjusted for sex and gestational age.

|  | Weight, grams  (95% confidence interval) | p |
| --- | --- | --- |
| Energy, 10 kcal/kg/d | 1.8 (0.8, 2.8) | 0.001 |
| Protein, g/kg/d | 5.4 (0.8, 9.9) | 0.02 |
| Fat, g/kg/d | 2.6 (1.2, 4.0) | <0.001 |
| Carbohydrate, g/kg/d | 1.3 (0.1, 2.5) | 0.04 |
