# Supplementary material for: Early postnatal nutrition after preterm birth and cardiometabolic risk factors in young adulthood
Source: PLoS One. 2018 Dec 28;13(12):e0209404. doi: 10.1371/journal.pone.0209404 (PMC6310277; doi:10.1371/journal.pone.0209404)
Supplement: S2 Table — Analysis of the first 3 weeks of life energy and macronutrient intakes of those born very low birth weight and cardiometabolic outcomes in adulthood, after exclution of those who recieved parenteral nutrition for more than one day or where born from a multiple pregnancy. (DOC) [file pone.0209404.s002.doc]

**S2 Table.** Sensitivity analysis. Effect of the first 3 weeks of life energy and macronutrient intake of VLBW infants on cardiometabolic outcomes in young adult life, by separate linear regression models adjusted for sex and age. Analysis after exclution of **(A)** those who recieved parenteral nutrition for more than one day (n=16, 13%, number of participants included to the analysis is 109) or **(B)** multiple pregnancies (n=21, 17%, number of participants included to the analysis is 104).

**S2 Table (A)**

|  | **Systolic blood pressure, mmHg** | **p** | **Diastolic blood pressure, mmHg** | **p** | **Fasting glucose, %** | **p** |
| --- | --- | --- | --- | --- | --- | --- |
| Energy, 10 kcal/kg/d | -0.60 (-2.12, 0.92) | 0.44 | -0.88 (-1.91, 0.15) | 0.09 | 0.79 (-0.16, 1.75) | 0.10 |
| Protein, g/kg/d | 3.31 (-2.87, 9.48) | 0.29 | 0.33 (-3.93, 4.59) | 0.88 | 3.94 (0.03, 8.01) | 0.05 |
| Fat, g/kg/d | 0.08 (-2.08, 2.25) | 0.94 | -0.58 (-2.06, 0.90) | 0.44 | 0.70 (-0.66, 2.08) | 0.31 |
| Carbohydrate, g/kg/d | -1.97 (-3.93, -0.02) | 0.05 | -1.37 (-2.71, -0.03) | 0.05 | 0.98 (-0.26, 2.24) | 0.12 |
|  | **2-hour glucose, %** | **p** | **Fasting insulin, %** | **p** | **2-hour insulin, %** | **p** |
| Energy, 10 kcal/kg/d | -0.37 (-3.03, 2.36) | 0.79 | 7.99 (1.47, 14.92) | 0.02 | 2.71 (-5.15, 11.23) | 0.51 |
| Protein, g/kg/d | 0.28 (-10.20, 11.99) | 0.96 | 36.55 (5.91, 76.04) | 0.02 | 8.86 (-21.37, 50.70) | 0.61 |
| Fat, g/kg/d | 0.45 (-3.35, 4.39) | 0.82 | 7.82 (-1.44, 17.96) | 0.10 | 2.57 (-8.43, 14.90) | 0.66 |
| Carbohydrate, g/kg/d | -1.80 (-5.20, 1.72) | 0.31 | 9.70 (1.09, 19.05) | 0.03 | -1.28 (-11.07, 9.57) | 0.81 |
|  | **Total cholesterol, mmol/L** | **p** | **HDL cholesterol, mmol/L** | **p** |  |  |
| Energy, 10 kcal/kg/d | -0.02 (-0.12, 0.09) | 0.77 | -0.04 (-0.09, 0.01) | 0.16 |  |  |
| Protein, g/kg/d | -0.03 (-0.45, 0.39) | 0.90 | -0.13 (-0.33, 0.08) | 0.23 |  |  |
| Fat, g/kg/d | 0.00 (-0.14, 0.15) | 0.97 | -0.04 (-0.11, 0.03) | 0.26 |  |  |
| Carbohydrate, g/kg/d | -0.05 (-0.19, 0.08) | 0.42 | -0.05 (-0.12, 0.02) | 0.14 |  |  |

**S2 Table (B)**

|  | **Systolic blood pressure, mmHg** | **p** | **Diastolic blood pressure, mmHg** | **p** | **Fasting glucose, %** | **p** |
| --- | --- | --- | --- | --- | --- | --- |
| Energy, 10 kcal/kg/d | 0.11 (-1.58, 1.81) | 0.89 | -0.49 (-1.66, 0.69) | 0.41 | 1.03 (0.02, 2.04) | 0.05 |
| Protein, g/kg/d | 4.60 (-2.46, 11.66) | 0.20 | 0.10 (-4.86, 5.06) | 0.97 | 3.80 (-0.51, 8.28) | 0.08 |
| Fat, g/kg/d | 0.84 (-1.58, 3.26) | 0.49 | -0.17 (-1.86, 1.52) | 0.85 | 0.95 (-0.51, 2.43) | 0.20 |
| Carbohydrate, g/kg/d | -0.70 (-2.70, 1.29) | 0.49 | -0.62 (-2.01, 0.77) | 0.38 | 1.14 (-0.05, 2.34) | 0.06 |
|  | **2-hour glucose, %** | **p** | **Fasting insulin, %** | **p** | **2-hour insulin, %** | **p** |
| Energy, 10 kcal/kg/d | -0.41 (-3.33, 2.60) | 0.78 | 11.32 (4.26, 18.85) | 0.002 | 4.30 (-3.99, 13.32) | 0.32 |
| Protein, g/kg/d | 0.75 (-11.13, 14.22) | 0.91 | 47.85 (11.80, 95.52) | 0.01 | 15.52 (-18.57, 63.88) | 0.41 |
| Fat, g/kg/d | 0.38 (-3.83, 4.77) | 0.86 | 10.99 (0.75, 22.26) | 0.03 | 4.34 (-7.39, 17.57) | 0.48 |
| Carbohydrate, g/kg/d | -1.73 (-5.11, 1.78) | 0.33 | 12.40 (3.96, 21.53) | 0.004 | -0.19 (-9.55, 10.14) | 0.97 |
|  | **Total cholesterol, mmol/L** | **p** | **HDL cholesterol, mmol/L** | **p** |  |  |
| Energy, 10 kcal/kg/d | 0.01 (-0.10, 0.12) | 0.87 | -0.02 (-0.08, 0.03) | 0.41 |  |  |
| Protein, g/kg/d | 0.00 (-0.46, 0.46) | 0.99 | -0.10 (-0.34, 0.13) | 0.37 |  |  |
| Fat, g/kg/d | 0.01 (-0.15, 0.16) | 0.94 | -0.03 (-0.11, 0.05) | 0.43 |  |  |
| Carbohydrate, g/kg/d | 0.03 (-0.10, 0.16) | 0.66 | -0.01 (-0.08, 0.05) | 0.71 |  |  |
