# Supplementary material for: Early postnatal nutrition after preterm birth and cardiometabolic risk factors in young adulthood
Source: PLoS One. 2018 Dec 28;13(12):e0209404. doi: 10.1371/journal.pone.0209404 (PMC6310277; doi:10.1371/journal.pone.0209404)
Supplement: S3 Table — Effect of early growth (weeks 1 to 3 and 4 to 6 of life) of very low birth weight and preterm infants on cardiometabolic outcomes in adulthood. (DOC) [file pone.0209404.s003.doc]

**S3 Table**. **Early growth and cardiometabolic outcomes in adulthood.** Effect of early growth (weeks 1 to 3 and 4 to 6 of life) of very low birth weight and preterm infants on cardiometabolic outcomes; **(A)** blood pressure and fasting glucose, **(B)** fasting insulin and glucose and insulin levels after 2-hour oral glucose tolerance test, and **(C)** cholesterol levels, in young adult life, by separate linear regression models.

**S3 Table** (A)

|  | **Systolic blood pressure, mmHg** | **p** | **Diastolic blood pressure, mmHg** | **p** | **Fasting glucose, %** | **p** |  |
| --- | --- | --- | --- | --- | --- | --- | --- |
| Weight, 10g, weeks 1-3, Model 1 | 0.50 (-1.89, 2.89) | 0.68 | 0.67 (-0.94, 2.27) | 0.41 | -0.64 (-2.03, 0.78) | 0.37 |  |
| Model 2 | 1.94 (-0.93, 4.80) | 0.18 | 1.56 (-0.35, 3.47) | 0.11 | -0.65 (-2.39, 1.13) | 0.47 |  |
| Weight, 10g, weeks 4-6, Model 1 | -0.08 (-0.27, 0.10) | 0.36 | -0.02 (-0.15, 0.11) | 0.76 | 0.05 (-0.06, 0.16) | 0.37 |  |
| Model 2 | -0.20 (-0.44, 0.04) | 0.10 | -0.07 (-0.23, 0.10) | 0.42 | 0.00 (-0.15, 0.14) | 0.95 |  |
| Length, 1SD, weeks 1-3, Model 1 | -0.70 (-3.24, 1.84) | 0.59 | -0.84 (-2.53, 0.84) | 0.32 | -0.21 (-1.76, 1.35) | 0.79 |  |
| Model 2 | 0.28 (-2.56, 3.12) | 0.84 | 0.10 (-1.80, 2.00) | 0.92 | 0.12 (-1.61, 1.88) | 0.89 |  |
| Length, 1SD, weeks 4-6, Model 1 | -0.38 (-4.08, 3.32) | 0.84 | 1.19 (-1.41, 3.79) | 0.37 | -0.21 (-2.57, 2.21) | 0.86 |  |
| Model 2 | 0.40 (-4.05, 4.85) | 0.86 | 2.07 (-1.06, 5.21) | 0.19 | -0.54 (-3.33, 2.34) | 0.71 |  |
| Head, 1SD, weeks 1-3, Model 1 | -0.40 (-2.58, 1.78) | 0.72 | 0.48 (-0.97, 1.93) | 0.51 | 0.46 (-0.83, 1.76) | 0.49 |  |
| Model 2 | 1.27 (-1.58, 4.12) | 0.38 | 1.72 (-0.16, 3.60) | 0.07 | 0.72 (-1.03, 2.50) | 0.42 |  |
| Head, 1SD, weeks 4-6, Model 1 | -4.78 (-7.84, -1.72) | 0.003 | -2.98 (-5.16, -0.81) | 0.01 | 0.68 (-1.29, 2.69) | 0.50 |  |
| Model 2 | -4.46 (-8.31, -0.60) | 0.02 | -2.27 (-5.06, 0.51) | 0.11 | 0.55 (-1.97, 3.14) | 0.67 |  |
|  |  |  |  |  |  |  |  |

**S3 Table (B)**

|  | **2-hour glucose, %** | **p** | **Fasting insulin, %** | **p** | **2-hour insulin, %** | **p** |
| --- | --- | --- | --- | --- | --- | --- |
| Weight, 10g, weeks 1-3, Model 1 | -0.64 (-2.03, 0.78) | 0.37 | 5.58 (-3.72, 15.78) | 0.25 | 4.59 (-6.69, 17.23) | 0.44 |
| Model 2 | 1.35 (-3.75, 6.71) | 0.61 | 3.15 (-7.80, 15.40) | 0.58 | 4.43 (-9.73, 20.82) | 0.56 |
| Weight, 10g, weeks 4-6, Model 1 | -0.01 (-0.32, 0.31) | 0.97 | 0.45 (-0.28, 1.19) | 0.22 | 0.58 (-0.30, 1.47) | 0.20 |
| Model 2 | -0.03 (-0.46, 0.40) | 0.89 | 0.51 (-0.43, 1.46) | 0.28 | 0.99 (-0.23, 2.22) | 0.11 |
| Length, 1SD, weeks 1-3. Model 1 | -1.59 (-5.94, 2.96) | 0.48 | 1.22 (-8.59, 12.09) | 0.81 | 6.05 (-6.45, 20.22) | 0.36 |
| Model 2 | -2.67 (-7.45, 2.35) | 0.29 | 3.45 (-7.30, 15.45) | 0.54 | 4.46 (-9.45, 20.52) | 0.55 |
| Length, 1SD, weeks 4-6, Model 1 | 0.87 (-5.62, 7.81) | 0.80 | 1.23 (-13.73, 18.79) | 0.88 | 6.90 (-11.74, 29.47) | 0.49 |
| Model 2 | 5,86 (-2.47, 14.89) | 0.17 | 12.78 (-6.16, 35.55) | 0.20 | 29.58 (2.57, 63.71) | 0.03 |
| Head, 1SD, weeks 1-3, Model 1 | 1.17 (-2.67, 5.17) | 0.55 | 6.01 (-2.50, 15.27) | 0.17 | 2.90 (-7.53, 14.51) | 0.60 |
| Model 2 | 0.22 (-4.77, 5.47) | 0.93 | 2.52 (-8.26, 14.58) | 0.66 | -2.45 (-15.57, 12.70) | 0.73 |
| Head, 1SD, weeks 4-6, Model 1 | -5.01 (-10.20, 0.47) | 0.07 | 6.45 (-6.69, 21.44) | 0.35 | 4.95 (-10.75, 23.41) | 0.56 |
| Model 2 | -4.84 (-11.53, 2.35) | 0.18 | 9.97 (-6.70, 29.62) | 0.25 | 13.49 (-8.11, 40.15) | 0.24 |

**S3 Table (C)**

|  | **Total cholesterol, mmol/L** | **p** | **HDL cholesterol, mmol/L** | **p** |  |  |
| --- | --- | --- | --- | --- | --- | --- |
| Weight, 10g, weeks 1-3, Model 1 | 0.01 (-0.15, 0.16) | 0.92 | 0.01 (-0.06, 0.09) | 0.77 |  |  |
| Model 2 | 0.12 (-0.08, 0.31) | 0.24 | 0.07 (-0.02, 0.16) | 0.14 |  |  |
| Weight, 10g, weeks 4-6, Model 1 | -0.01 (-0.02, 0.01) | 0.30 | -0.00 (-0.01, 0.00) | 0.36 |  |  |
| Model 2 | 0.00 (-0.01, 0.02) | 0.77 | 0.00 (-0.01, 0.01) | 0.49 |  |  |
| Length, 1SD, weeks 1-3, Model 1 | -0.05 (-0.22, 0.12) | 0.53 | -0.02 (-0.10, 0.07) | 0.72 |  |  |
| Model 2 | 0.01 (-0.18, 0.21 | 0.90 | -0.02 (-0.11, 0.07) | 0.67 |  |  |
| Length, 1SD, weeks 4-6, Model 1 | -0.11 (-0.37, 0.16) | 0.43 | 0.00 (-0.12, 0.12) | 0.95 |  |  |
| Model 2 | 0.02 (-0.31, 0.36) | 0.89 | 0.12 (-0.02, 0.27) | 0.10 |  |  |
| Head, 1SD, weeks 1-3, Model 1 | -0.04 (-0.19, 0.11) | 0.59 | -0.03 (-0.10, 0.04) | 0.42 |  |  |
| Model 2 | 0.06 (-0.13, 0.26) | 0.53 | 0.02 (-0.08, 0.11) | 0.71 |  |  |
| Head, 1SD, weeks 4-6, Model 1 | -0.30 (-0.52, -0.07) | 0.01 | -0.04 (-0.15, 0.06) | 0.40 |  |  |
| Model 2 | -0.27 (-0.57, 0.02) | 0.07 | 0.03 (-0.10, 0.16) | 0.66 |  |  |

The values are unstandardized regression coefficients, (95% confidence interval), adjusted for sex and age (Model 1). Model 2 is adjusted with sex, age, gestational age, birth weight SD score, highest parental education, maternal smoking during pregnancy, maternal preeclampsia, postnatal characteristics (neonatal exposure of treatment with ventilator (days), bronchopulmonary dysplasia, septicemia and persistent ductus arteriosus), adult body fat percentage, leisure-time exercise intensity and smoking status. Weight analysis included 121 participants in weeks 1-3 and 114 in weeks 3-6. Length analysis included 120 participants in weeks 1-3 and 112 participants in weeks 4-6. Head circumference analysis included 113 participants in weeks 1-3 and 110 participants in weeks 4-6.
